# Significance of Liver Zonation in Hepatocellular Carcinoma

**DOI:** 10.3389/fcell.2022.806408

**Published:** 2022-06-23

**Authors:** Shizhe Yu, Jie Gao, Haoren Wang, Long Liu, Xudong Liu, Yuantong Xu, Jihua Shi, Wenzhi Guo, Shuijun Zhang

**Affiliations:** ^1^ Department of Hepatobiliary and Pancreatic Surgery, The First Affiliated Hospital of Zhengzhou University, Zhengzhou, China; ^2^ Henan Engineering Technology Research Center for Organ Transplantation, Zhengzhou, China; ^3^ Open and Key Laboratory for Hepatobiliary & Pancreatic Surgery and Digestive Organ Transplantation at Henan Universities, Zhengzhou, China; ^4^ Department of Oncology, The First Affiliated Hospital of Zhengzhou University, Zhengzhou, China; ^5^ Department of Hepatopancreatobiliary Surgery, The First People’s Hospital of Kunming, Calmette Hospital, Kunming, China

**Keywords:** hepatocellular carcinoma, liver zonation, tumor dedifferentiation, single-cell RNA sequence, multiplex immunofluorescence, PON1, FTCD, ALAD

## Abstract

Liver zonation is fundamental to normal liver function, and numerous studies have investigated the microstructure of normal liver lobules. However, only a few studies have explored the zonation signature in hepatocellular carcinoma (HCC). In this study, we investigated the significance of liver zonation in HCC with the help of single-cell RNA sequencing (scRNA-seq) and multicolor immunofluorescence staining. Liver zonation-related genes were extracted from the literature, and a three-gene model was established for HCC prognosis. The model reliability was validated using bulk RNA and single-cell RNA-level data, and the underlying biological mechanism was revealed by a functional enrichment analysis. The results showed that the signaling pathways of high-risk groups were similar to those of perivenous zones in the normal liver, indicating the possible regulating role of hypoxia in HCC zonation. Furthermore, the co-staining results showed that the low-grade tumors lost their zonation features whereas the high-grade tumors lost the expression of zonation-related genes, which supported the results obtained from the sequencing data.

## Introduction

The liver is the central metabolic organ. It performs various critical functions that maintain body homeostasis. It also produces a considerable proportion of circulating proteins (Gebhardt, 1992; Trefts et al., 2017; Ben-Moshe et al., 2019; Ben-Moshe and Itzkovitz, 2019). After the development of scRNA-seq and spatial transcriptomes, numerous studies have researched the micro-anatomical structure of the normal liver (Halpern et al., 2017, 2018; Kietzmann, 2017; Ben-Moshe et al., 2019; Ben-Moshe and Itzkovitz, 2019; Gola et al., 2021). Approximately 50% of hepatocytic genes are expressed in a zoned manner. These genes are responsible for most liver-specific functions, such as albumin synthesis, drug metabolism, glycolipid metabolism, free radical scavenging, and immune activity (Ben-Moshe et al., 2019). Different subsets of hepatocytes perform various liver activities, and this optimization of function mainly depends on liver zonation (Jungermann, 1986; Meijer et al., 1990; Bartl et al., 2015). Besides their distinct gene expression profiles, hepatocytes in different lobular regions also have different epigenetic characteristics, regenerative capacities, susceptibility to damage, and other functional aspects (Dezső et al., 2017; Brosch et al., 2018; Wei et al., 2021). It is well known that the liver is the only organ with the ability to regenerate itself, but not all hepatocytes have the ability to proliferate (Michalopoulos and Bhushan, 2021). Recent studies have shown that only hepatocytes at a specific zonation can self-replicate in the presence of pathological damage to the liver (He et al., 2021; Wei et al., 2021), which suggests that not all hepatocytes have the same potential to develop into tumor cells (Sia et al., 2017). However, few studies have explored the variation in liver zonation characteristics in hepatocellular carcinoma (HCC).

In this study, we combined large sample transcriptome cohorts, single-cell sequencing data, and multiplex immunofluorescence techniques to explore the liver zonation-related genes in HCC. We found that liver zonation-related genes are liver-specific and commonly downregulated in HCCs. The liver zonation-related signature (LZRS) is a reliable predictor of an HCC patient’s prognosis and can identify the more malignant tumor cell subtypes at the single-cell resolution level. These signature genes decreased with the activation of the proto-oncogene network in HCC cells and were negatively correlated with the degree of HCC dedifferentiation. Although highly differentiated HCCs still have characteristic genes, they are not zoned and are expressed in a mixed fashion. In contrast, low-differentiated tumors lose the expression of characteristic genes. Our findings provide a framework to further understand the changing landscape of liver zonation during the development of HCC.

## Materials and Methods

### Public Data Collection and Processing  

The normalized gene-level RNA-seq data and clinical information from 364 patient TCGA-LIHC cohorts were downloaded from UCSC Xena (https://xenabrowser.net/) using the R package UCSC Xena Tools (Wang and Liu, 2019). The LIRI-JP validation sets for 258 patients and GSE14520 validation sets for 239 patients were obtained by downloading the RNA-seq data and the related clinicopathological data from the International Cancer Genome Consortium (ICGC) website (https://dcc.icgc.org/projects/LIRI-JP) (Zhang et al., 2019) and the Gene Expression Omnibus (GEO, https://www.ncbi.nlm.nih.gov/geo/). The single-cell RNA sequencing barcode sequences and raw gene expression matrix were downloaded from CNP0000650 (Sun et al., 2021). Mutation data that contained somatic variants were stored in the Mutation Annotation Format (MAF) form and were downloaded from the Genomic Data Commons (GDC) (https://portal.gdc.cancer.gov).

### Exploring the Expression Patterns of Liver Zonation-Related Genes in HCC

**FIGURE 1:**
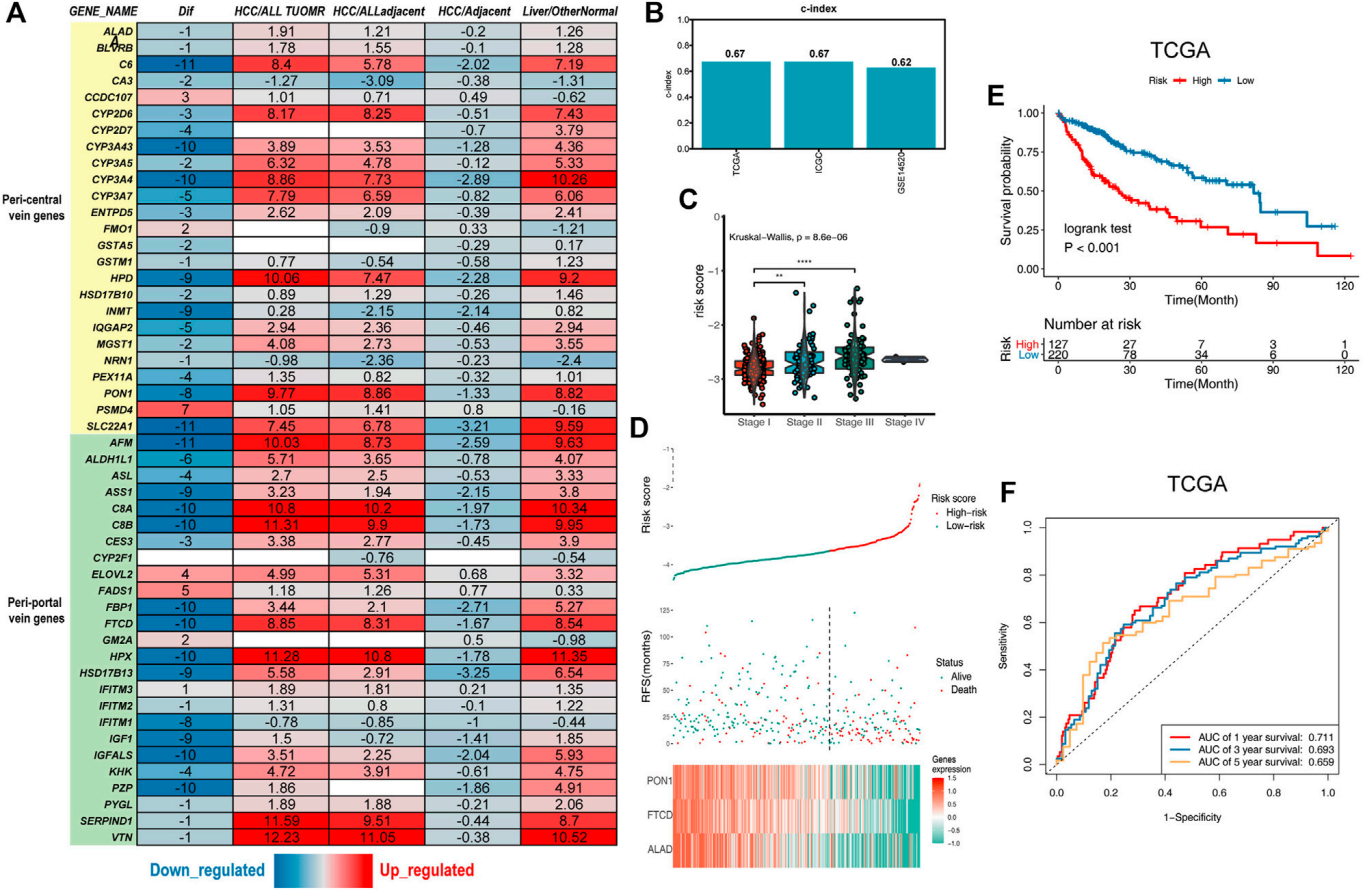
Establishment of the liver zonation-related prognostic signature. **(A)** Summary of liver zonation-related genes. Dif: The number of differentially expressed datasets. Red/blue for consensus upregulated/downregulated. HCC/AllTumor: red/blue for the positive/negative fold change in log2 scale by comparing HCC with all tumors (TCGA data). HCC/AllAdjacent: red/blue for the positive/negative fold change in log2 scale by comparing HCC with all adjacent samples (TCGA data). HCC/Adjacent: red/blue for the positive/negative fold change in log2 scale by comparing HCC with adjacent samples (HCCDB data). Liver/OtherNormal: red/blue for the positive/negative fold change in log2 scale by comparing liver with normal tissues (GTEx and TCGA data). **(B)** C-index of the three-gene signature was 0.67 in the TCGA cohort, 0.67 in the ICGC cohort, and 0.62 in the GSE14520 cohort. **(C)** Violin diagram showing higher risk scores for the higher tumor stage. **(D)** Top graphs show the distribution of risk scores; the center graphs show the survival status of patients in the training cohorts; the bottom graphs show expression patterns of the three genes.**(E)** Kaplan–Meier plot of the three-gene signature in TCGA cohort. **(F)** tROC curve of the three-gene signature in TCGA.

The liver zonation-related genes (*n* = 50, Figure 1A) were selected from previously published articles and further verified using the Human Protein Atlas (Uhlén et al., 2015; Halpern et al., 2017, 2018; Ben-Moshe et al., 2019; Droin et al., 2021). Furthermore, the expression patterns of liver zonation-related genes were summarized using the HCCDB database. We used the 4D metric defined by a previous study to summarize the patterns (Lian et al., 2018). In detail, 4D metrics, which include four metrics, are defined in the following way:

1) The liver-specific metric quantifies the specificity of a gene in the liver compared to other tissues:

$$\log_{2}FC_{1}=\left(\log_{2}{\left(\frac{{\bar{x}}_{liver}}{{\bar{\bar{x}}}_{tissue}}\right)}_{GTE_{x}}+\log_{2}{\left(\frac{{\bar{x}}_{adjacent\;of\;LIHC}}{{\bar{\bar{x}}}_{adjacent}}\right)}_{TCGA}\right)/2.$$

2) The deregulation metric measures the deregulation extent of a gene in HCCs compared to adjacent samples:

$$\log_{2}FC_{2}=\bar{\log_{2}{\left(\frac{{\bar{x}}_{HCC}}{{\bar{x}}_{adjacent}}\right)}_{HCCDB}}.$$

3) The tumor-specific metric quantifies the specificity of a gene in HCCs compared to other tissues:

$$\log_{2}FC_{3}=\log_{2}{\left(\frac{{\bar{x}}_{HCC}}{{\bar{\bar{x}}}_{adjacent}}\right)}_{TCGA}.$$

4) The HCC-specific metric is the specificity of a gene in HCCs compared to other tumor types:

$$\log_{2}FC_{4}=\log_{2}{\left(\frac{{\bar{x}}_{HCC}}{{\bar{x}}_{tumor}}\right)}_{TCGA}.$$

### Development and Validation of the Liver Zonation-Related Signature for HCC

Cases from the TCGA datasets were used as the training cohort to establish the liver zonation-related signature (LZRS).

The signature generation procedure was as follows: 1) a univariate Cox regression identified prognostic liver zonation-related genes in the TCGA-LIHC cohort. 2) Then, a LASSO regression was performed on the prognostic genes to the fit prediction models in the TCGA-LIHC cohort. 3) The model was detected in two validation datasets (GSE14520 and ICGC-LIHC).

The risk score for each patient was calculated by the LASSO model weighting coefficient as follows:

$$riskscores=\sum_{i=1}^{n}Coe\;f_{j}*X_{j},$$

where *n* represents the number of key genes, *Coef*_*j*_ is the LASSO coefficient of gene *j*, and *X*_*j*_ is the normalized expression value of gene *j* (formimidoyltransferase cyclodeaminase [FTCD]: −0.0522, aminolevulinate dehydratase [ALAD]: −0.0136, and paraoxonase 1 [PON1]: −0.0247). Then, the concordance c-index proposed by Harrell was applied to validate the predictive ability of the signature in all datasets using the “survcomp” R package (Harrell, 1982; Haibe-Kains et al., 2008). A larger c-index indicated that the predictive ability of the model was more accurate.

### Processing of Single-Cell RNA-Seq Data

Single-cell RNA sequencing data were processed for dimension reduction and unsupervised clustering by following the workflow in Seurat (v. 4.0.2) (Butler et al., 2018). In brief, first, the read counts for each cell were divided by the total counts for that cell and multiplied by the scale factor (10,000), and then natural log transformed. A principal component analysis (PCA) matrix with 50 components was calculated to reveal the main axes of variation, and the data were denoised by using the “RunPCA” function with the default parameter. For visualization, the dimensionality of each dataset was further reduced using uniform manifold approximation and projection (UMAP) implemented in the “RunUMAP” function (Becht et al., 2019). We retained cell clustering based on a previous study (Sun et al., 2021). The cluster-specific marker genes were identified by using the “FindAllMarkers” function with the MAST algorithm (Finak et al., 2015).

The liver zonation-related feature scores were calculated by the negative LASSO model weighting coefficient:

$$featurescores=\sum_{i=1}^{n}Coe\;f_{j}*X_{j},$$

where *n* represents the number of key genes, *Coef*_*j*_ is the LASSO coefficient of gene *j*, and *X*_*j*_ is the normalized expression value of gene *j* (FTCD: −0.0522, ALAD: −0.0136, and PON1: −0.0247).

### Survival Analysis

The malignancy of different tumor cell subpopulations in the scRNA-seq data was identified by extracting the top 10 differentially expressed genes (DEGs) in each cluster, and then, the potential prognostic significance of these genes was assessed using the LIHC data from GEPIA2 (http://gepia2.cancer-pku.cn/#index).

The Kaplan–Meier curves were also generated to graphically demonstrate the overall survival (OS) of the high-risk and low-risk groups, which were stratified by the liver zonation-related signature. The R package “survminer” was utilized to perform the survival analysis, and the optimal cut-off was ascertained by the “surv_cutpoint” function.

### Bioinformatics Analysis

Gene set enrichment analysis (GSEA) was further used to investigate the functional enrichment of risk score-associated genes using the R package “clusterProfiler” (Yu et al., 2012). The Benjamini–Hochberg method was used to adjust the nominal *p*-values (false discovery rate, FDR) for multiple testing. A gene set variation analysis (GSVA) was performed to evaluate the pathway activities in the scRNA-seq data and bulk data. A single-sample gene set enrichment analysis (ssGSEA) implemented in the R package GSVA was used to quantify the relative infiltration of 28 immune cells in the TCGA-LIHC cohort. The gene sets used in the enrichment analysis were downloaded from the Molecular Signature Database (MsigDB, http://software.broadinstitute.org/gsea/msigdb/).

### Tissue Samples

The tumor samples were collected from HCC patients at the First Affiliated Hospital of Zhengzhou University, China. They consisted of 136 paired samples of primary HCC tumor and paracancerous tissues from January 2014 to August 2019, each with a follow-up of more than 2 years. This study complies with the guidelines of the China Ethical Committee and the Helsinki Declaration. Informed consent was obtained.

The tissues were fixed with formalin, embedded in paraffin, and arranged into three tissue chips.

### Multicolor Immunofluorescence

Tissue sections were dewaxed in xylene overnight and rehydrated in a graded alcohol series (ethanol:deionized water 100:0, 90:10, 80:20, 70:30, 50:50, and 0:100; 5 min each). After deparaffinization with xylene and rehydration, antigen retrieval was performed by microwave treatment in 10 mmol sodium citrate buffer (pH 6.0) for 20 min. The endogenous peroxidase was blocked with 3% H_2_O_2_ in methanol, and non-specific binding was blocked for 10 min using a protein-blocking buffer. The sections were washed in phosphate-buffered saline (PBS). In a microwave oven, heat-induced epitope retrieval was conducted in Tris-EDTA buffer at pH 9 for 25 min, and then, the sample was allowed to cool down to 25°C. The endogenous peroxidase activity was blocked by incubating the slides in 3% hydrogen peroxide for 25 min and then blocked with 3% bovine serum albumin (BSA) in Tris-buffered saline (TBS) for 30 min.

The ALAD (Abcam, ab151754), PON1 (Proteintech, 18155-1-AP), and FTCD (Proteintech, 21959-1-AP) antibodies were labeled with Alexa Fluor® 488, 555, and 647, respectively, using Lightning-Link Rapid Kits (Abcam, ab236553, ab269820, and ab269823, respectively). The primary antibodies were incubated at 4°C overnight. After overnight incubation at 4°C, the sections were washed with PBS and stained with DAPI.

Whole slide digital images were scanned using a Pannoramic DESK scanner (3DHISTECH), and all IF staining were quantified by QuPath software (Bankhead et al., 2017).

### Statistical Analysis

Student’s *t*-test was conducted to make the statistical comparison, and the “pheatmap” R package was used to generate heatmaps. Survival analysis was conducted using the Kaplan–Meier method, and the prediction performance of the risk model was evaluated using the receiver operating characteristic (ROC) in the “time-ROC” R package. Multivariate Cox regression analyses were used to investigate the prognostic value of the risk score. The hazard ratio (HR) and 95% confidence intervals (CI) for each variable were also calculated where needed. *p* < 0.05 was defined as a statistically significant difference. All of our analyses were conducted by R software version 4.0.2 (https://www.r-project.org).

## Results

### Summary and Characterization of Liver Zonation-Related Genes

We used zonation-related genes in normal liver lobules obtained from a literature review to obtain a representative list of liver zonation genes (Halpern et al., 2017; Halpern et al., 2018; Ben-Moshe et al., 2019; Droin et al., 2021). These genes are robust according to several different experimental platforms, including single-cell RNA sequencing (scRNA-seq), spatial transcriptomes, seqFISH, and spatial sorting proteomics (Halpern et al., 2017; Halpern et al., 2018; Ben-Moshe et al., 2019). Liver zonation-related genes can be divided into two main groups: peri-central vein genes and peri-portal vein genes (Ben-Moshe and Itzkovitz, 2019). These genes are controlled by factors such as oxygen, nutrients, and microorganisms and form the basis for normal liver function. To explore the liver zonation characteristics in HCC, we first investigated the expression of these genes in HCC and its paraneoplastic tissues in 15 HCC transcriptomic datasets (Lian et al., 2018). These genes had the highest expression levels in normal liver tissues. They were downregulated in cancerous liver tissues but were still higher than in other non-hepatic tissues, indicating their specificity in liver tissues (Figure 1A).

### Construction and Validation of a Three-Gene Zonation-Related Signature

To obtain the prognostic genes, we retained the genes significantly associated with prognosis by univariate Cox analysis. The results of the univariate Cox regression analysis of 24 genes were used in the LASSO regression to identify robust markers in the TCGA-LIHC cohort (Ally et al., 2017). PON1, FTCD, and ALAD were 445 extracted and had the most significant HCC overall 446 survival times (Figure 1D, Supplementary Figures S1A,B). The ICGC dataset and GSE14520 were used as external validation cohorts to verify the predictive ability of the model (Zhang et al., 2019). There was a significant difference in survival among patients in the high- and low-risk groups within the three cohorts (Figures 1E,F; Supplementary Figures S1C–F), and the risk scores were upregulated with the increasing TNM stage (Figure 1C). The credibility of this model was validated by assessing the C-index in the three cohorts (0.67, 0.67, and 0.62, respectively) (Figure 1B).

### Three-Gene Zonation-Related Signature Could Accurately Identify More Malignant Cells at the Single-Cell Level

**FIGURE 2:**
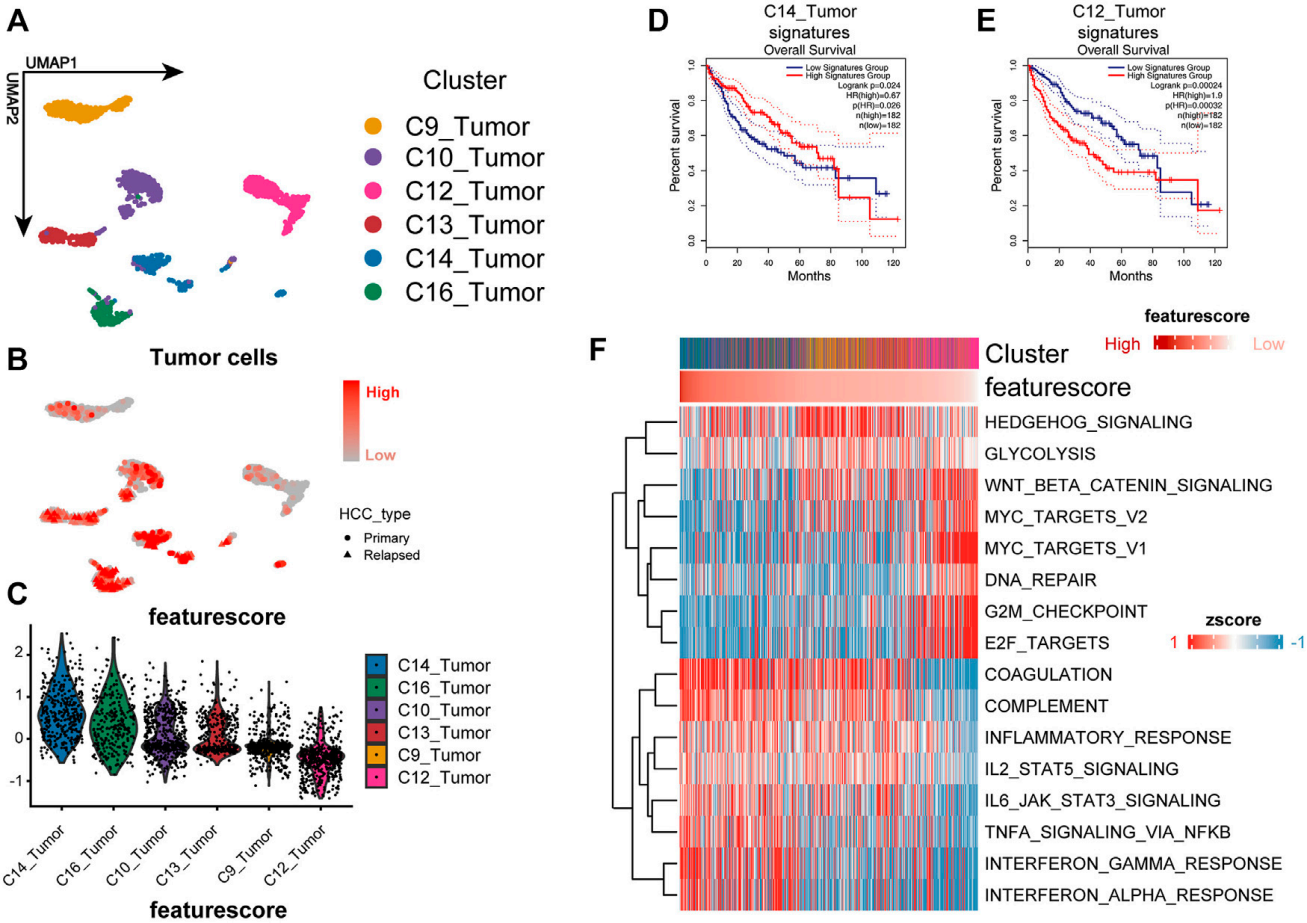
Three-gene signature could identify more malignant cells well in the single-cell level. **(A)** UMAP plot shows the cluster of tumor cells. The annotation of cell types follows the original authors. **(B,C)** Feature plot and violin plot show the feature score of each cluster. The higher the feature score, the less malignant is the tumor. **(D,E)** Kaplan–Meier plot of the C14_Tumor signature and C12_Tumor signature in TCGA cohort. **(F)** Heat map shows the GSVA enrichment of each cell; cells are sorted according to the feature score.

The performance of the model was validated by the SMART-seq2–based high-quality scRNA-seq data for HCC (Figure 2A) (Sun et al., 2021). We calculated the feature score for each tumor cell by taking the negative value of the risk score. Cluster C14 had the highest feature score, and cluster C12 had the lowest feature score (Figures 2B,C). Differential genes were calculated for each cluster using the MAST algorithm (Finak et al., 2015). The top 10 differential genes were selected as the cluster-specific signatures for the survival analysis to assess the degree of malignancy for each cluster. We compared whether there was a survival difference between the two groups to determine the malignancy of the malignant cell clusters. Signature C14 represented a better prognosis, and signature C12 showed a worse prognosis (Figures 2D,E). In contrast, the other cluster signatures were not related to a prognosis, indicating that the model could accurately identify the more malignant cells.

### Loss of Liver Zonation Features Is Associated With Proto-Oncogene Network Activation and Tumor Cell Immune Escape

**FIGURE 3:**
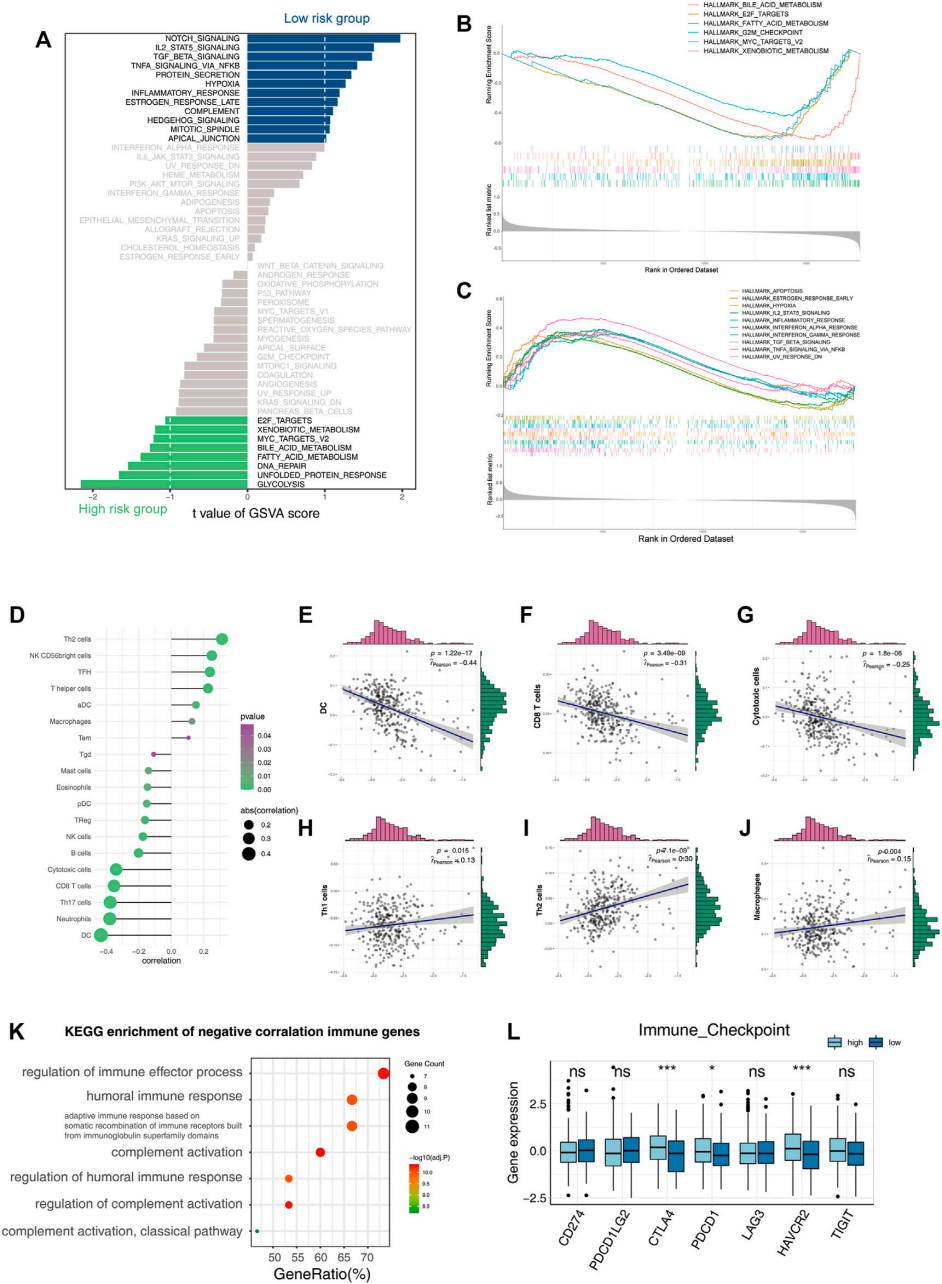
Functional enrichment analysis of the three-gene signature. **(A)** Bar plot of GSVA enrichment in the high-risk group and low-risk group. **(B,C)** GSEA enrichment results in the high-risk group and low-risk group. **(D–J)** Correlation of the risk score with infiltrative immune cells. **(K)** KEGG enrichment result of immune genes’ negative correlation with risk scores. **(L)** Boxplot shows the expression of immune checkpoints in the high-risk group and low-risk group (*p < 0.05, **p < 0.01, and ***p < 0.001).

To investigate the biological mechanisms underlying the loss of liver zonation-related features leading to a poor prognosis for HCC, we performed a GSVA on each cell and ranked the cells from the highest to the lowest according to the feature score (Hänzelmann et al., 2013) (Figure 2F). In general, the malignancy of HCC cells gradually increases as the feature score decreases in three significant ways: 1) the proliferation and activation of the proto-oncogene network (“G2M checkpoint,” “WNT/*β*-catenin signaling,” “MYC targets,” and “E2F targets”); 2) the loss of intrinsic hepatic features (“coagulation” and “complement”); and 3) the downregulation of the inflammatory response (“inflammatory response,” “IL2_STAT5 signaling,” “IL6_JAK_STAT3 signaling,” “TNF*α* signaling *via* NF-κB,” “interferon *α* response,” and “interferon *γ* response”). The upregulation of the “Hedgehog” and “WNT/*β*-catenin” signaling pathways and the metabolic pathways, such as glycolysis, in HCC were consistent with the variation in typical liver zonation (Rebouissou et al., 2016). They are the most relevant pathways for early liver cancer progression (Benhamouche et al., 2006; Sia et al., 2017; Perugorria et al., 2019). Notably, the bulk level analysis was consistent with the single-cell level analysis. We performed a GSVA on the TCGA cohort and calculated the differential pathways between high- and low-risk groups using the limma package (Ritchie et al., 2015). The high-risk group had a greater proliferative capacity and glycolytic activity, while the low-risk group had a more potent immune activation profile (Figure 3A). The GSEA results also confirmed this discovery (Figures 3B,C). These results suggest that the LZRS can be a good marker for predicting early proto-oncogenic pathway activation in HCC.

The immune microenvironment of tumors is associated with their prognosis (Thorsson et al., 2018). Notably, both the bulk-level and single-cell resolution data showed negative correlations between tumor inflammation levels and the LZRS (Figure 2F, Figures 3A–C).

Deconvolution analysis of the tumor microenvironment showed that antitumor immune cells, such as CD8, CTL, B cells, and Th17, were negatively correlated with the LZRS, indicating poor immune infiltration in the high-risk group (Figures 3D–J). A total of 2,498 immune-related genes were extracted from the ImmPort database. We calculated the Pearson correlations between these genes and their risk score and selected immune genes that were negatively correlated with the risk score (*r* < −0.3 and *p* < 0.05). A KEGG pathway enrichment analysis showed that these genes were mainly involved in the immune activation process (Figure 3K). We examined the expression of classical immune checkpoints in the high- and low-risk groups; CTLA4, PDCD1, and HAVCR2 were significantly upregulated in the high-risk group (Figure 3L). This implies that immune infiltration may be an important cause of prognostic differences.

### Multiplex Immunofluorescence Reveals the Alteration and Loss of Typical Zonation Characteristics in HCC

**FIGURE 4:**
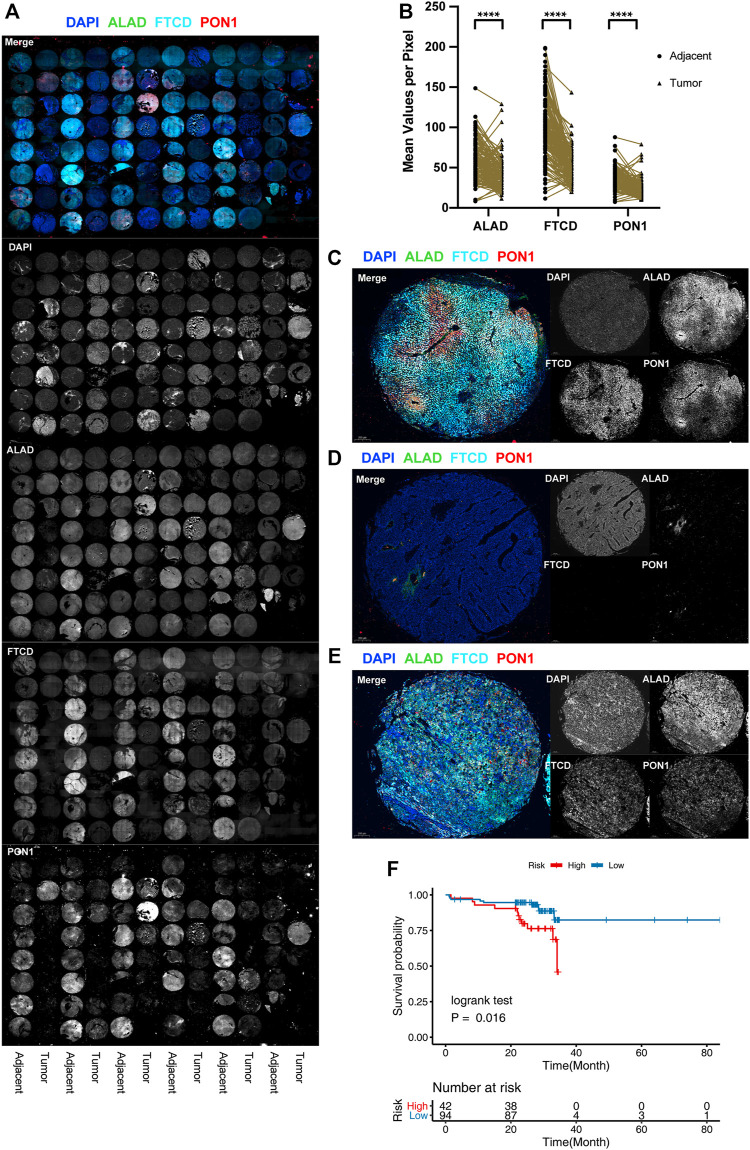
Protein level validation for external cohorts. **(A)** Full tissue microarray scans with nuclei labeled with DAPI (blue), ALAD labeled with Alexa Fluor 488, FTCD labeled with Alexa Fluor 550, and PON1 labeled with Cy5. For better visualization, FTCD signals are converted to pseudo-color. **(B)** Distribution of the difference in staining intensities of ALAD, FTCD, and PON1 in HCC tissues compared with that in paired adjacent tissues. (****p < 0.001). **(C–E)** Representative images of multicolor IF staining in tissues. Adjacent tissues **(C)**, triple-negative tumor tissues **(D)**, and triple-positive tumor tissues with chaotic distribution **(E)**. **(F)** K-M plot of the three-gene prognosis model in 136 patient external validation cohorts.

To determine the protein expression of three genes, we performed multicolor IF staining using 136 paired samples of primary HCC tumor and paracancerous tissues from January 2014 to August 2019, each with a follow-up of more than 2 years. The patient characteristics are listed in Supplementary Table S1. The genes were mainly expressed in adjacent tissues, but they were absent in tumor tissues (Figures 4A,B). Co-staining revealed that the normal tissues showed distinct zonation, the low-grade tumors lost their zonation, and the high-grade tumors showed no expression of these zonation-related genes (Figures 4C–E). Considering that liver function is mainly based on zonation, the alteration and loss of normal zonation characteristics represent the degree of tumor cell dedifferentiation. We divided the samples based on the calculated risk score for each sample. The results showed there were significant survival differences between the high- and low-risk groups (Figure 4F), and the validation results were consistent with the results of the study analysis.

## Discussion

The liver exhibits a profound division of labor between hepatocytes residing in different regions of the liver, and such a division of labor is fundamental if the liver is to perform its normal functions (Jungermann, 1986; Jungermann and Keitzmann, 1996; Ben-Moshe and Itzkovitz, 2019; Gola et al., 2021). Recent studies have suggested that in addition to the functional differences, hepatocytes in different regions exhibit different responses to injury in pathological situations because only some of them can reproduce (Michalopoulos and Bhushan, 2021, 2021; Wei et al., 2021). Therefore, understanding and modeling the changes in the liver during disease progression require the characterization of hepatocyte function at each lobular coordinate. This study combined machine learning, single-cell sequencing, and multiplex IF approaches to extract signatures from liver zonation-related genes, most of which were associated with HCC prognosis, and determined the changes in liver zonation characteristics during HCC progression.

Our machine learning results showed that PON1, FTCD, and ALAD best responded to the changing characteristics of zonation during HCC progression. Paraoxonase 1 is a hydrolase located on HDL and has been postulated to have a protective effect on low-density lipoprotein oxidation (Mackness and Mackness, 2015). Previous studies have reported that PON1 is significantly upregulated during the regulation of chronic liver disease and plays an active role in oxidative stress, fibrosis, and hepatocyte apoptosis (Ferré et al., 2006). The FTCD encoded by this gene is a bifunctional enzyme that channels 1-carbon units from formiminoglutamate, a metabolite in the histidine degradation pathway, to the folate pool (Kanarek et al., 2018). The ALAD enzyme is composed of eight identical subunits and catalyzes the condensation of two molecules of delta-aminolevulinate to form porphobilinogen (Sassa, 1998). All three proteins are liver-specific and expressed at high levels. They showed significant downregulation at the RNA and protein levels in HCC.

Sorting tumor cells according to their feature score in the single-cell dataset revealed that the degree of HCC dedifferentiation progressively increased with the decreasing expression of these three genes. Our multiplex IF results also supported the conclusion that the highly differentiated HCC tissue still expresses these genes but loses zonation. In contrast, the hypodifferentiated HCC tissue completely lost the expression of these genes. On the one hand, it shows that this signature can be used to determine the degree of HCC differentiation and to assess the prognosis of patients. On the other hand, it suggests that the expression of these genes may be involved in the dedifferentiation of tumor cells.

The origin of HCCs remains a mystery (Sia et al., 2017). Previous studies have speculated that they originate from liver progenitor cells, but there is still no direct evidence for this speculation (Mokkapati et al., 2014). However, recent studies have found that only some regions of the hepatocytes can regenerate and participate in repairing the liver after injury and that these cells may be the origin of hepatocarcinogenesis (He et al., 2021; Wei et al., 2021). Once again, this shows the importance of the intrinsic zonation of the liver in liver cancer. The upregulation of the “Hedgehog” and “WNT/*β*-catenin” signaling pathways and metabolic pathways, such as glycolysis, in HCCs was consistent with the variation in typical liver zonation. Probably due to hypoxia, the metabolic and related regulation pathways in high-risk groups were similar to those in the perivenous zone of a normal liver. Previous studies proposed that the oxygen gradient was a regulator of liver zonation, where the low oxygen content in the perivenous zone would activate the *β*-catenin signaling pathway *via* the hypoxia-inducible factor (HIF) system (Matsumura and Thurman, 1983; Wolfle et al., 1983; Kietzmann, 2017). In this concept, gradients of morphogens, such as “WNT/*β*-catenin” and “Hedgehog,” restrict the gene expression to differentiated hepatocytes located in specific zones of the liver acinus (Benhamouche et al., 2006; Sekine et al., 2006; Lade and Monga, 2011; Kietzmann, 2017; Perugorria et al., 2019). The HCC cells that can adapt to the hypoxic environment are more likely to originate from the periportal zones.

There were several limitations to this study. The LZRS model can be reproduced using a simple immunohistochemistry assay, making it attractive for clinical translation and implementation. Although the clinical significance of the LZRS in HCC is promising, researchers should acknowledge some limitations. First, all of the samples from this study were retrospective, and future validation of the LZRS should be performed using prospective multicenter cohort studies. Second, there was a lack of single-cell sequencing datasets that explored advanced liver disease and early HCC, as well as focused on the changes in the hepatocytes themselves during liver disease. This made it difficult to determine the role of the LZRS in the hepatocarcinogenesis process. Third, the cause of liver lobular zonation disorder during the progression of chronic liver disease is unclear, and further *in vivo* and *in vitro* experiments need to be undertaken.

In summary, we showed that the characteristics of liver zonation were disrupted in low-grade HCC tissues and vanished in high-grade HCC tissues, representing a loss and dedifferentiation of liver features. Our results show that zonation-related genes can accurately classify patients into different risk groups and predict immunotherapy efficacy.

## Data Availability

The datasets presented in this study can be found in online repositories. The names of the repository/repositories and accession number(s) can be found in the article.

## Supplementary Material

Supplementary Figure S1 Validation of the liver zonation-related prognostic signature. **(A,B)** LASSO regression analysis determined the three-gene signature for OS prediction. **(A)** Cross-validation plot shows the confidence intervals for each lambda variable; **(B)** shows the trajectory of each independent variable, and the horizontal axis represents the log value of the independent variable lambda, while the vertical axis represents the coefficient of the independent variable. **(C,D)** Kaplan–Meier plot in ICGC cohort **(C)** and GSE14520 cohort **(D)**. **(E,F)** tROC curve of 25-gene signature in TCGA cohort **(E)** and ICGC cohort **(F)** **(G)**. Multivariate Cox regression analysis of clinical parameters and risk scores for OS.
