# Supplementary figures and images for: Significance of Liver Zonation in Hepatocellular Carcinoma
Source: Front Cell Dev Biol. 2022 Jun 23;10:806408. doi: 10.3389/fcell.2022.806408 (PMC9260020; doi:10.3389/fcell.2022.806408)

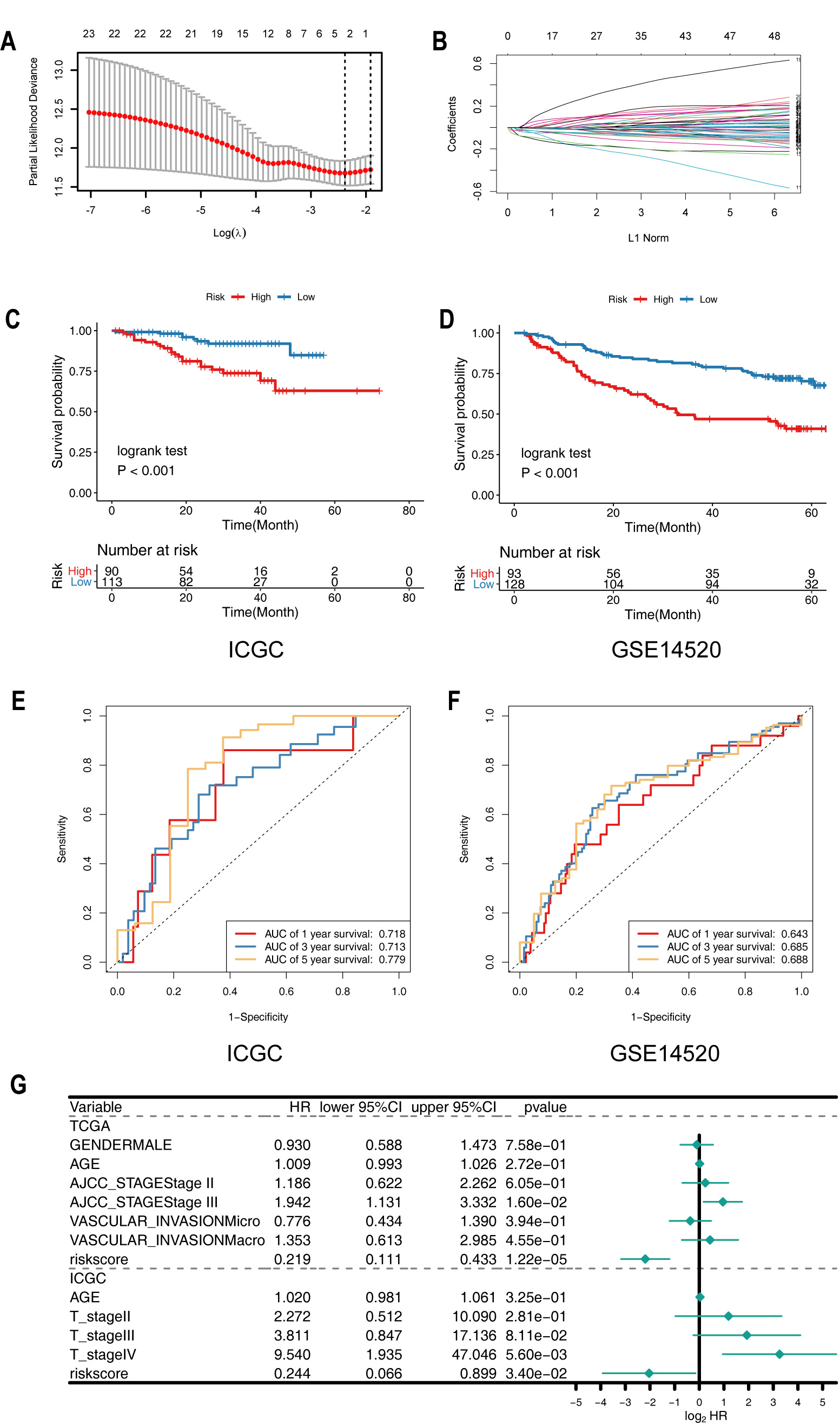

Supplement: Supplementary file 1 [file Image1.jpeg]
